# CLIP-RL: Closed-Loop Video Inpainting with Detection-Guided Reinforcement Learning

**DOI:** 10.3390/s26020447

**Published:** 2026-01-09

**Authors:** Meng Wang, Jing Ren, Bing Wang, Xueping Tang

**Affiliations:** Faculty of Information Engineering and Automation, Kunming University of Science and Technology, 727 South Jingming Road, Kunming 650500, China; renjing@stu.kust.edu.cn (J.R.); 20232204231@stu.kust.edu.cn (B.W.); tangxueping@stu.kust.edu.cn (X.T.)

**Keywords:** video inpainting, reinforcement learning, inpainting detection, closed-loop framework, temporal consistency

## Abstract

Existing video inpainting methods typically combine optical flow propagation with Transformer architectures, achieving promising inpainting results. However, they lack adaptive inpainting strategy optimization in diverse scenarios, and struggle to capture high-level temporal semantics, causing temporal inconsistencies and quality degradation. To address these challenges, we make one of the first attempts to introduce reinforcement learning into the video inpainting domain, establishing a closed-loop framework named CLIP-RL that enables adaptive strategy optimization. Specifically, video inpainting is reformulated as an agent–environment interaction, where the inpainting module functions as the agent’s execution component, and a pre-trained inpainting detection module provides real-time quality feedback. Guided by a policy network and a composite reward function that incorporates a weighted temporal alignment loss, the agent dynamically selects actions to adjust the inpainting strategy and iteratively refines the inpainting results. Compared to ProPainter, CLIP-RL improves PSNR from 34.43 to 34.67 and SSIM from 0.974 to 0.986 on the YouTube-VOS dataset. Qualitative analysis demonstrates that CLIP-RL excels in detail preservation and artifact suppression, validating its superiority in video inpainting tasks.

## 1. Introduction

**Figure 1:**
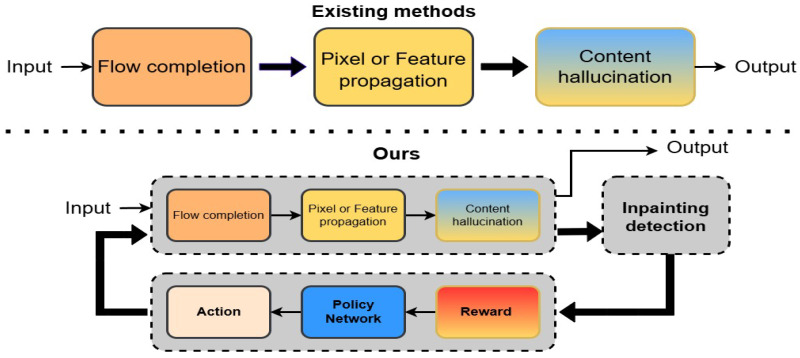
Overall pipeline comparison between existing methods and this method.

Video inpainting (VI) reconstructs missing video regions, ensuring visual and temporal coherence, and is vital in multimedia computing applications like object removal [1,2], damaged region restoration [3,4], watermark removal [5], and film post-production [6]. For instance, VI enhances film authenticity by seamlessly removing safety cables or props. Hybrid frameworks combining optical flow propagation [7,8,9] and video Transformers [5,7,10] excel in static or small-occlusion scenarios but struggle with dynamic backgrounds or large occlusions due to limited adaptability. This results in suboptimal inpainting performance, limiting their applicability in complex scenarios. To address these challenges, we propose a closed-loop inpainting framework based on reinforcement learning CLIP-RL, which uses an inpainting detection module to improve inpainting performance and efficiency in complex scenarios, as shown in Figure 1.

**Figure 2:**
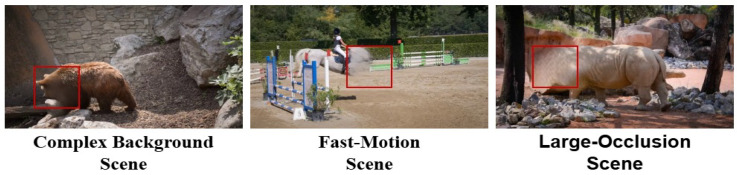
Typical failure cases of existing video inpainting models. The red boxes indicate areas with poor inpainting results.

Traditional video inpainting methods rely on fixed strategies, which struggle to adapt to scene-specific characteristics such as dynamic backgrounds and long-term temporal dependencies. Transformer-based models, such as [5,11], often underperform in complex scenarios, constrained by the limited representational capacity of standard self-attention heads. This limitation restricts their ability to capture motion correlations across distant frames, leading to blurry outputs and visual artifacts. As shown in Figure 2, these models often struggle in challenging scenarios—they fail to maintain motion consistency for fast-moving objects and to handle large occlusions, where large-scale motion and missing regions remain problematic. In complex backgrounds, the inpainting results become unstable, often exhibiting motion blur, incomplete object recovery, and inconsistent background reconstructions. Moreover, maintaining temporal coherence remains a key challenge, as methods relying on pixel-level optical flow alignment [2,5,12] often suffer from accumulated flow estimation errors under large occlusions or rapid motion, leading to inter-frame jitter and visual artifacts. Although recent advancements incorporate inertial priors to improve flow estimation in dynamic scenes [9], they still struggle to capture high-level semantic information, limiting their ability to address cumulative alignment errors. Consequently, these factors collectively degrade inpainting quality and hinder the broader adoption of video inpainting in real-world applications.

To address the limitations of traditional video inpainting methods in maintaining temporal consistency and robustness under complex scenarios, inspired by Ukwuoma et al. [13], this study proposes a novel video inpainting framework that pioneers the integration of reinforcement learning (RL) and inpainting detection, overcoming the constraints of fixed-strategy approaches. Unlike generative adversarial networks (GANs) [14], our framework models the inpainting module as the action component of an RL agent, with the inpainting detection module serving as the environment, generating predicted masks to identify low-quality inpainted regions. The RL module employs a policy network and reward function to dynamically refine the inpainting strategy. Additionally, we introduce a weighted temporal alignment loss, combining latent space alignment and detection feedback, to mitigate optical flow error accumulation and enhance temporal consistency of inpainted videos. Experiments on the YouTube-VOS [15] and DAVIS [16] datasets validate the framework’s effectiveness, achieving 0.69% and 1.2% improvements in PSNR and SSIM, respectively, and lower LPIPS compared to that of the baseline ProPainter [11], yielding more natural inpainting results. Ablation studies further confirm the critical roles of the inpainting detection module, RL module, and weighted temporal alignment loss, highlighting the superiority of adaptive inpainting strategies in complex scenarios. The main contributions of this study include the following:

1. We introduce a Reinforcement Learning-Based Video Inpainting Framework CLIP-RL. To our knowledge, we make one of the first attempts to integrate reinforcement learning and inpainting detection in a closed-loop setting for video inpainting. This module generates predicted masks to identify low-quality inpainted regions, providing real-time feedback to guide the agent in dynamically adjusting its inpainting strategy.
2. We design a Multi-Dimensional Action Space. The action space (i) adjusts the number of Transformer attention heads to balance accuracy and efficiency, (ii) optimizes the fusion strength between inpainted and original frames for smooth boundary transitions, and (iii) dynamically tunes reward weights to adapt the optimization objective to scene complexity.
3. We design a Weighted Temporal Alignment Loss. By leveraging latent space alignment and feedback from the inpainting detection module, this loss mitigates the accumulation of optical flow errors in fast-motion scenes. Combined with the reinforcement learning framework, it enhances temporal consistency and inpainting quality in complex dynamic scenarios.

## 2. Related Work

Video inpainting (VI) aims to fill missing regions while restoring spatiotemporal consistency. Contemporary methods can be grouped into three paradigms—flow-guided, Transformer-based, and hybrid approaches that combine the two. Despite their strengths in spatiotemporal modeling, most existing pipelines use static inpainting strategies and operate in an open-loop manner (i.e., without closed-loop feedback for iterative refinement), which limits robustness in dynamic scenes and under large occlusions.

### 2.1. Existing Video Inpainting Methods

Flow-guided methods utilize inter-frame motion information to fill missing regions, serving as a core technique in video inpainting [17,18,19]. However, in scenarios involving complex nonlinear motion or prolonged occlusions, optical flow estimation is susceptible to errors, leading to temporal inconsistencies in repaired regions. Wang et al. [20] employ FlowNet to extract local and non-local flow features, combined with edge completion techniques, to enhance detail inpainting and improve repair quality in long-term occlusion scenarios. Similarly, IGFC [9] introduces an inertial prior, assuming relatively stable motion trends within local temporal windows, to generate accurate flow estimates through reference frame alignment. By using dilated convolutions [21], IGFC expands the receptive field, significantly improving spatiotemporal consistency in complex motion scenarios. Nevertheless, in long-sequence videos or large missing regions, flow-guided methods degrade significantly due to unreliable flow information.

Transformer-based methods, leveraging their superior global context modeling capabilities [22], excel in handling large missing regions and have become a research hotspot. FuseFormer [10] employs soft segmentation to divide video frame features into overlapping patches, utilizing inter-patch information fusion to achieve cross-frame content propagation and inpainting. DLFormer [23] proposes a content hallucination approach based on a discrete latent space, encoding video frames into latent representations and using Transformers to infer missing regions, effectively avoiding blurriness and distortion caused by inadequate continuity modeling. However, the high computational complexity and memory demands of Transformer architectures limit their practical deployment in resource-constrained environments.

Hybrid frameworks integrate flow estimation with Transformer architectures to balance precise local detail inpainting and global spatiotemporal consistency. For instance, FGT+ [12] introduces a flow-guided deformable attention mechanism, constructing cross-frame motion propagation chains through a bidirectional flow completion network. It employs a temporal deformable multi-head attention module to dynamically adjust query positions based on flow offsets. Experiments demonstrate that this method achieves strong performance on the DAVIS dataset while maintaining a computational cost of 488.59 GFLOPs. However, its static fusion strategy (e.g., a fixed flow-attention blending weight of 0.7) lacks adaptive adjustment and dynamic feedback mechanisms, limiting performance improvements in dynamic scenes. The static nature of these methods struggles to address the diversity of complex video scenarios, motivating our exploration of reinforcement learning-based dynamic optimization strategies with closed-loop feedback for adaptive inpainting.

### 2.2. Dynamic Optimization and Feedback

Reinforcement learning (RL) has demonstrated remarkable dynamic optimization capabilities in computer vision tasks. Pirinen et al. [24] proposed a deep RL-based region proposal network (drL-RPN), integrating sequential decision-making into region selection to enhance accuracy and robustness in object detection. Similarly, Ren et al. [25] applied a deep RL framework with an actor-critic approach to image captioning, dynamically adjusting generation strategies to improve adaptability in complex visual environments. These studies indicate that RL, through sequential decision-making and environmental feedback, can outperform purely supervised approaches in certain dynamic tasks. Inspired by this, we introduce reinforcement learning into video inpainting and propose the CLIP-RL framework, aiming to address the limitation of traditional models that lack adaptive adjustment of inpainting strategies.

Inpainting detection techniques, designed to identify manipulated regions in videos, hold significant value in network security and copyright protection. FOCAL [26] extracts inpainting features via pixel-level contrastive learning and employs unsupervised HDBSCAN [27] clustering to dynamically distinguish manipulated from authentic regions, enhancing detection accuracy and generalization. GIID-Net [28] adopts a three-stage architecture (augmentation, feature extraction, decision), integrating pre-filters and attention mechanisms to achieve precise detection of inpainting artifacts, demonstrating generalization to unseen inpainting methods. Despite these advancements, the application of inpainting detection in video inpainting remains underexplored. Inpainting detection can provide real-time feedback signals to guide dynamic optimization, but effectively integrating it with inpainting to form a closed-loop mechanism remains a key challenge.

To address these limitations, we draw inspiration from Ukwuoma et al. [13], who proposed an RL-guided image inpainting framework combining an Autoencoder (AE), a Latent Generative Adversarial Network (GAN) with attention mechanisms, and a reinforcement learning (RL) agent. The RL agent selects the optimal latent vector from the GAN to generate a clean global feature vector (GFV) for reconstruction. Trained with a composite reward function incorporating Chamfer, GFV, and discriminator losses, their RLG-Net enhances image completion and classification performance even with 70% missing data. We adopt their approach as a conceptual baseline because it effectively integrates RL with generative modeling, providing a strong foundation for learning-based inpainting. However, their method focuses on static images without considering temporal dependencies. Building on this insight, we extend RL-based inpainting to videos through a closed-loop system that integrates detection and inpainting modules, allowing the RL agent to adaptively maintain spatio-temporal consistency and robustness.

## 3. The Proposed CLIP-RL Approach

**Figure 3:**
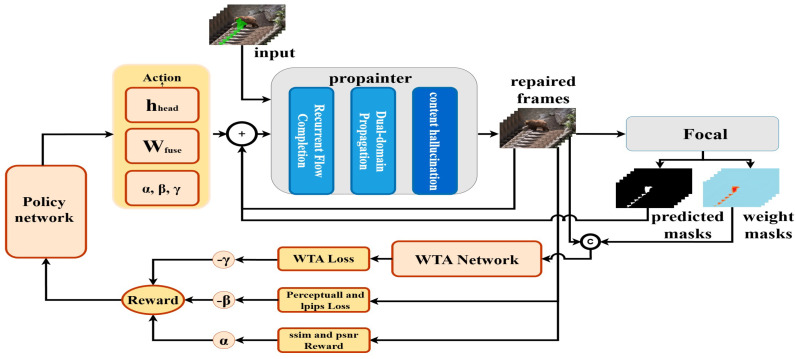
The CLIP-RL framework integrates video inpainting and inpainting detection via reinforcement learning. The ProPainter module performs inpainting, guided by feedback masks from the inpainting detection module. A reward function with weighted terms drives policy updates, with the action space adjusting Transformer attention heads, feature fusion weights, and reward weights. Arrows indicate the iterative agent-environment feedback loop.

Given a corrupted video sequence $X={\left\{X_{t}\in R^{H\times W\times 3}\right\}}_{t=1}^{T}$, where *T* denotes the number of video frames and $X_{t}$ represents the *t*-th frame, along with a corresponding binary mask sequence $M={\left\{M_{t}^{0}\in R^{H\times W\times 1}\right\}}_{t=1}^{T}$, where each $M_{t}^{0}$ indicates the initial missing regions in the *t*-th frame, the goal of video inpainting is to reconstruct a video sequence $\hat{Y}={\left\{{\hat{Y}}_{t}^{N}\in R^{H\times W\times 3}\right\}}_{t=1}^{T}$ with consistent spatial structures and temporal coherence in the missing regions. Existing inpainting models typically employ static strategies, which exhibit limited robustness across diverse scene inpainting tasks. To address this limitation, we propose a novel reinforcement learning-based video inpainting framework as shown in Figure 3. This framework enhances inpainting quality and cross-scene adaptability through adaptive optimization. The implementation details of the proposed framework are elaborated below.

### 3.1. Reinforcement Learning-Driven Closed-Loop Inpainting Framework

To enhance the quality and cross-scene robustness of video inpainting, this paper proposes a dynamic closed-loop inpainting framework based on reinforcement learning, integrating an inpainting module with an inpainting detection module to form an optimization loop of inpainting-detection-feedback-refinement. The inpainting module repairs damaged video frames, and the results are evaluated by the inpainting detection module, which generates predicted masks to identify areas with suboptimal inpainting quality. These masks are fed back to the agent, enabling the reinforcement learning module to adjust the inpainting strategy iteratively based on a reward function. The reward function combines PSNR, SSIM, perceptual loss ($L_{\mathrm{Perce}}$), LPIPS loss ($L_{\mathrm{LPIPS}}$), and weighted temporal alignment loss ($L_{\mathrm{WTA}}$) to assess inpainting quality comprehensively.

#### 3.1.1. Interaction Between Inpainting and Inpainting Detection Modules

The core task of the agent is to adjust the inpainting strategy based on environmental feedback. The inpainting module, built upon the ProPainter model [11], leverages dual-domain propagation and sparse Transformer mechanisms to efficiently fill missing regions in videos while maintaining spatiotemporal consistency. The module takes the initial mask $M_{t}^{0}$ and the corrupted video frame $X_{t}$ as inputs. Subsequent predicted masks, generated by the inpainting detection module, provide feedback for inpainting.

First, the module estimates inter-frame motion using optical flow to ensure consistency. Then, it propagates pixel and feature information from adjacent frames to the missing regions. A sparse Transformer-based content hallucination module synthesizes visual content for the unrecovered regions. The output of the inpainting module is defined as:

(1) $${\hat{Y}}_{t}^{n}=A\;\left(M_{t}^{n-1},\;X_{t-i:t+i},\;{\hat{Y}}_{t}^{n-1};\;h_{\mathrm{head}}^{n-1}\right)$$

where A denotes the inpainting function, generating the inpainted frame ${\hat{Y}}_{t}^{n}\in R^{H\times W\times 3}$. Here, ${\hat{Y}}_{t}^{n}$ represents the *t*-th frame after the *n*-th inpainting iteration, where n=1,2,…,N is the iteration index and t=1,2,…,T is the frame index. $M_{t}^{n-1}$ is the predicted mask for the *t*-th frame after the (n−1)-th iteration. $X_{t-i:t+i}$ represents the sequence of reference frames, where *i* denotes the temporal window radius, and ${\hat{Y}}_{t}^{0}=X_{t}$ is the initial corrupted frame. The dynamic adjustment of attention heads $h_{\mathrm{head}}^{n-1}$, introduced in Section 3.1.3, optimizes the Transformer’s multi-head attention mechanism during inpainting.

The inpainting detection module plays a critical role in our framework by providing feedback signals for refinement. We adopt the pre-trained FOCAL model [26] as the detection backbone due to its strong generalization ability and fine-grained localization performance. Unlike conventional classification-based detectors that rely on global supervision, FOCAL leverages pixel-level contrastive learning and image-wise unsupervised clustering, capturing the relative differences between inpainted and pristine regions within each image. This formulation enables robust detection of subtle texture inconsistencies and boundary artifacts—features highly analogous to distortions introduced by video inpainting—making FOCAL particularly suitable for our task. To further enhance detection accuracy and mitigate potential sensitivity to detection errors, we provide the initial ground-truth mask $M_{t}^{0}$ as an auxiliary input to the FOCAL model. This guidance constrains the detector’s attention to inpainted regions, suppressing false positives in unaltered areas and reducing unnecessary computation. By incorporating region-level priors, the detection module achieves more stable and reliable responses across consecutive frames, preventing the accumulation of false detections during iterative refinement. The output of the detection module is defined as:

(2) $$\left(M_{t}^{n},\;D_{t}^{n}\right)=f_{\det}\left(M_{t}^{0},\;{\hat{Y}}_{t}^{n}\right)$$

where $f_{\det}$ is the detection function of the FOCAL model, $M_{t}^{n}\in R^{H\times W\times 1}$ is the predicted mask for the *t*-th frame after the *n*-th iteration, indicating low-quality inpainting regions, and $D_{t}^{n}\in R^{H\times W\times 3}$ is the weight mask for the *t*-th frame after the *n*-th iteration, providing supervisory information for the weighted temporal-alignment loss ($L_{\mathrm{WTA}}$).

The agent interacts with the environment via a reinforcement-learning framework, adjusting the inpainting strategy to maximize the reward function. The reward function not only incorporates traditional inpainting-quality metrics (e.g., PSNR, SSIM) but also includes perceptual loss ($L_{\mathrm{Perce}}$), LPIPS loss ($L_{\mathrm{LPIPS}}$), and weighted temporal-alignment loss ($L_{\mathrm{WTA}}$) to further optimize spatiotemporal consistency.

#### 3.1.2. Reward Function Design and Policy Network Optimization

The CLIP-RL framework optimizes video inpainting strategies through the synergistic interaction of a reward function and a policy network. The reward function evaluates the inpainting outcomes and provides feedback, while the policy network generates optimized inpainting actions based on this feedback. This section elaborates in detail on the design of the reward function and the optimization process of the policy network.

1. Reward Function Design with Weighted Temporal Alignment Loss

**Figure 4:**
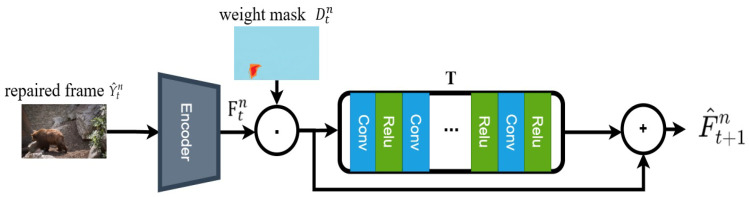
Weighted Temporal Alignment Network.

To enhance temporal consistency, we propose the Weighted Temporal Alignment Loss ($L_{\mathrm{WTA}}$), leveraging latent space alignment, an adaptive alignment module, and feedback from inpainting detection to mitigate motion blur and artifacts. Specifically, $L_{\mathrm{WTA}}$ maps the restored frame ${\hat{Y}}_{t}^{n}$ (the *t*-th frame in the *n*-th iteration) to the latent space through a pretrained encoder *E*, extracting high-level semantic features $F_{t}^{n}=E\left({\hat{Y}}_{t}^{n}\right)$. The latent representation $F_{t}^{n}$ captures global semantic information rather than being limited to local pixel similarity. The temporal alignment module $T(F_{t}^{n},D_{t}^{n})$ outputs the aligned features for the next frame:

(3) $${\hat{F}}_{t+1}^{n}=T\left(F_{t}^{n},D_{t}^{n}\right)$$

where $D_{t}^{n}$ is the weight mask generated by the inpainting detection module, providing additional supervisory information to guide the alignment module in focusing on inpainted regions. The weighted temporal alignment loss $L_{\mathrm{WTA}}^{n}$ is defined as:

(4) $$L_{\mathrm{WTA}}^{n}=\frac{1}{T-1}\sum_{t=1}^{T-1}{∥F_{t+1}-{\hat{F}}_{t+1}^{n}∥}_{1}$$

where $F_{t+1}$ represents the true latent features of the next frame, and ${∥\cdot ∥}_{1}$ denotes the L1 norm, measuring inter-frame feature consistency. By minimizing $L_{\mathrm{WTA}}^{n}$, temporal discontinuities and artifacts are effectively reduced, ensuring temporal coherence in the inpainting results (see Figure 4, Weighted Temporal Alignment Network).

To comprehensively optimize inpainting quality, the reward function integrates both positive and negative metrics. The positive metrics employ Structural Similarity Index SSIM and Peak Signal-to-Noise Ratio PSNR, where the former ensures global consistency in brightness, contrast, and structure, the latter measures pixel-level accuracy:

(5) $$Q_{s}^{n}=\frac{1}{T}\sum_{t=1}^{T}\mathrm{SSIM}\left({\hat{Y}}_{t}^{n}\right)$$

(6) $$Q_{p}^{n}=\frac{1}{T}\sum_{t=1}^{T}\mathrm{PSNR}\left({\hat{Y}}_{t}^{n}\right)$$

Negative metrics incorporate perceptual loss $L_{\mathrm{Perce}}^{n}$ and Learned Perceptual Image Patch Similarity loss $L_{\mathrm{LPIPS}}^{n}$, which maintain semantic and structural consistency in the feature space, improving visual naturalness and the quality of complex textures. The final reward signal is defined as:

(7) $$R^{n}=\alpha \cdot \left(Q_{s}^{n}+Q_{p}^{n}\right)-\beta \cdot \left(L_{\mathrm{Perce}}^{n}+L_{\mathrm{LPIPS}}^{n}\right)-\gamma \cdot L_{\mathrm{WTA}}^{n}$$

where $R^{n}$ represents the reward value for the *n*-th iteration, and α, β, and γ are dynamically adjusted weight coefficients during the RL process to balance the importance of each metric.

2. Policy Network Optimization

Based on the optimization of the reward function, the policy network generates inpainting actions. The policy network takes the state $s^{n}$ as input, which comprises the inpainted frames and the predicted masks output by the inpainting detection module. Specifically, given the state $s^{n}$,

(8) $$s^{n}=\left[{\hat{Y}}^{n},M^{n}\right]$$

where ${\hat{Y}}^{n}={\left\{{\hat{Y}}_{t}^{n}\in R^{H\times W\times 3}\right\}}_{t=1}^{T}$ represents the sequence of inpainted video frames in the *n*-th iteration, and $M^{n}={\left\{M_{t}^{n}\in R^{H\times W\times 1}\right\}}_{t=1}^{T}$ denotes the sequence of predicted masks generated by the inpainting detection module in the *n*-th iteration. The policy network π, optimized via the Proximal Policy Optimization (PPO) algorithm, maps the input state $s^{n}$ to a continuous action space governing the inpainting strategy. To facilitate effective exploration and optimization within this continuous manifold, the network parameterizes the action distribution by outputting the mean μ and standard deviation σ of a Gaussian distribution for each dimension. During the training phase, actions are stochastically sampled from $N(\mu ,\sigma^{2})$ to enable the agent to explore diverse strategies. In the inference phase, the mean μ is adopted deterministically to ensure stable and reproducible inpainting results. The final action vector is thus derived as:

(9) $$A^{n}=\pi \left(s^{n}\right)$$

where $A^{n}$ represents the inpainting actions generated based on the input state $s^{n}$, with n∈{1,2,…,N}, indicating the *n*-th inpainting iteration, and *N* being the maximum number of inpainting iterations. To ensure that the output actions meet the requirements of the inpainting task, a dynamic constraint Clip is applied to each dimension of the output actions.

#### 3.1.3. Adaptive Action-Space Design

In the CLIP-RL framework, the action space *A* is central to the agent’s optimization of the inpainting strategy. The agent selects the optimal action $A^{n}$ based on the current state $s^{n}$, enabling adaptive video inpainting. This work designs an action space *A* that dynamically adjusts the number of attention heads ($h_{\mathrm{head}}$), feature-fusion strength ($w_{\mathrm{fuse}}$), and reward weights (α,β,γ), achieving fine-grained control and global optimization of the inpainting model. Each dimension of the action space is constrained with a Clip function, ensuring the output actions align with the video inpainting task requirements, balancing quality and efficiency. The action space is defined as:

(10) $$A=(h_{\mathrm{head}},\;w_{\mathrm{fuse}},\;\alpha ,\;\beta ,\;\gamma )$$

where $h_{\mathrm{head}}$ represents the number of attention heads, $w_{\mathrm{fuse}}$ denotes the feature-fusion strength, and α,β,γ are the weights of the reward-function components.

1. Dynamic Adjustment of Attention Heads $h_{\mathrm{head}}$

To balance the computational overhead introduced by the reinforcement learning process while enhance the model’s inpainting capability, we design an action that dynamically adjusts the number of attention heads. In the Transformer model, the number of attention heads $h_{\mathrm{head}}$ determines the granularity of feature interaction. For complex scenarios such as large missing areas or dynamic backgrounds, increasing $h_{\mathrm{head}}$ enhances global context modeling and captures more details. In simpler static scenes, reducing $h_{\mathrm{head}}$ lowers computational complexity and improves efficiency. The policy network predicts the optimal number of attention heads based on the current state $s^{n}$, and dynamically applies this action to the inpainting operator A.

(11) $$h_{\mathrm{head}}^{n}=\mathrm{Round}\;\left(\mathrm{Clip}\;\left(\pi_{\mathrm{head}}\left(s^{n}\right),\;2,\;12\right)\right)$$

(12) $${\hat{Y}}_{t}^{n+1}=A\;\left(M_{t}^{n},\;X_{t-i:t+i},\;{\hat{Y}}_{t}^{n};\;h_{\mathrm{head}}^{n}\right)$$

where $\pi_{\mathrm{head}}\left(s^{n}\right)$ is the output of the policy network for the state $s^{n}$, and the Clip function ensures that $h_{\mathrm{head}}^{n}$ lies within the range [2,12]. The Round(·) operation discretizes it to the nearest integer to ensure a valid number of attention heads. The predicted $h_{\mathrm{head}}^{n}$ is then injected into the inpainting operator A(·) in Equation (12) as a control parameter, enabling the Transformer to adaptively adjust the number of active heads according to scene complexity.

2. Dynamic Control of Feature-Fusion Strength $w_{\mathrm{fuse}}$

In video inpainting tasks, the boundary transition between the restored region and the original region is key to ensuring visual consistency. Traditional “hard replacement” strategies directly cover the missing regions, which can easily lead to boundary artifacts, color deviations, and visual discontinuities in complex dynamic scenes. To address this issue, this paper proposes a feature fusion strategy based on reinforcement learning, which dynamically adjusts the fusion strength $w_{\mathrm{fuse}}$ to achieve smooth fusion between the restored and original frames. The policy network predicts $w_{\mathrm{fuse}}$ as follows:

(13) $$w_{\mathrm{fuse}}^{n}=\mathrm{Clip}\;\left(\pi_{\mathrm{fuse}}\left(s^{n}\right),\;0.5,\;0.8\right)$$

where $\pi_{\mathrm{fuse}}\left(s^{n}\right)$ is the output of the policy network for the state $s^{n}$, and the Clip function ensures that $w_{\mathrm{fuse}}^{n}$ lies within the range [0.5,0.8]. In regions with large missing areas, $w_{\mathrm{fuse}}^{n}$ is increased to enhance global consistency; while in detail-rich areas, $w_{\mathrm{fuse}}^{n}$ is decreased to preserve more original information.

While feature fusion effectively mitigates boundary discontinuities, it inherently carries a risk of over-smoothing in high-frequency regions. To prevent such blurring artifacts, our framework leverages the composite reward function, which integrates blur-sensitive Perceptual and LPIPS losses. These metrics serve as critical constraints during optimization, implicitly penalizing texture degradation caused by excessive fusion. Consequently, the agent learns to dynamically attenuate $w_{\mathrm{fuse}}$ in detail-rich areas, ensuring that boundary smoothing is applied selectively to maintain global consistency without compromising local high-fidelity details.

3. Dynamic Balancing of Reward Weights α,β,γ

To adapt to diverse inpainting scenarios, this paper proposes dynamically adjusting the reward weights α,β,γ. This mechanism, through the policy network, dynamically generates the weight coefficients α, β, and γ based on the current environment state $s^{n}$ to balance the relative importance of each component in the reward function. Specifically, α controls the weights of SSIM and PSNR, increasing for fine-grained inpainting scenes to improve visual quality. β controls the weights of $L_{\mathrm{Perce}}^{n}$ and $L_{\mathrm{LPIPS}\;}^{n}$, increasing for regions with complex textures or heavy occlusion to better capture details and visual consistency. γ controls the weight of $L_{\mathrm{WTA}}$, increasing for dynamic backgrounds or fast motion scenes to ensure frame consistency:

(14) $$\left\{\begin{array}{cc} \alpha^{n} & =\mathrm{Clip}\;\left(\pi_{\alpha }\left(s^{n}\right),\;0.8,\;1.0\right),\\ \beta^{n} & =\mathrm{Clip}\;\left(\pi_{\beta }\left(s^{n}\right),\;0.3,\;0.6\right),\\ \gamma^{n} & =\mathrm{Clip}\;\left(\pi_{\gamma }\left(s^{n}\right),\;0.1,\;0.3\right), \end{array}\right.$$

where $\pi_{\alpha }\left(s^{n}\right)$, $\pi_{\beta }\left(s^{n}\right)$, and $\pi_{\gamma }\left(s^{n}\right)$ are the outputs of the policy network based on the current state $s^{n}$, and the Clip function ensures that the weight values are constrained within reasonable ranges.

To mitigate potential reward bias—where the agent may overemphasize easily optimized objectives such as PSNR or SSIM—the training process adopts Proximal Policy Optimization (PPO) with a clipped surrogate objective and entropy regularization. The clipping mechanism constrains policy updates within a trust region, ensuring stable learning dynamics, while entropy regularization encourages continued exploration over diverse reward combinations, thereby preventing convergence toward any single objective.

## 4. Results and Discussion

### 4.1. Experimental Setup

To validate the effectiveness of the proposed method, experiments were conducted on the YouTube-VOS [15] and DAVIS [16] datasets. The YouTube-VOS [15] dataset comprises 4453 natural scene videos, with 3471 training used for network training and 508 test videos for performance evaluation. The DAVIS [16] dataset provides 150 videos with high-precision annotations, of which 50 videos are used for quantitative analysis and 100 for ablation studies.

A two-stage progressive training strategy was adopted based on the YouTube-VOS [15] training set. In the first stage, the initial 1000 videos were selected for pre-training the inpainting module, while the inpainting detection module was independently trained following the official training protocol described in FOCAL [26]. Specifically, the module was optimized using a pixel-level contrastive loss on the constructed pseudo-inpainting dataset, which enables it to learn discriminative features for identifying manipulated regions. We adopted the same data augmentation strategies as the original FOCAL implementation to ensure robust and stable detection capabilities before integrating it into the closed-loop system. In the second stage, reinforcement learning was performed using the proximal policy optimization (PPO) algorithm with the remaining videos. This stage aimed to enhance inpainting quality, temporal consistency, and robustness in complex video scenarios. To improve training stability, an experience replay buffer with 50,000 trajectories and an entropy regularization mechanism were introduced to prevent premature convergence. Additionally, the policy network employed a periodic synchronous update mechanism to ensure stable and continuous improvement during training.

During evaluation, the proposed method was compared with several state-of-the-art (SOTA) methods, such as STTN [29] and FuseFormer [10]. Quantitative evaluation was conducted using PSNR, SSIM, and LPIPS metrics to demonstrate the superiority of the proposed method in video inpainting tasks.

### 4.2. Quantitative Evaluation

To assess the effectiveness of our proposed video inpainting framework, we conducted quantitative comparisons with several advanced video inpainting methods, including VINet [17], DFGVI [2], CPN [4], OPN [30], 3DGC [31], STTN [29], FGVC [7], TSAM [19], FFM [10], FGT [8], LNFVI [20] and ProPainter [11]. As shown in Table 1, our method demonstrates superior inpainting performance on the YouTube-VOS and DAVIS datasets, achieving the best results across multiple evaluation metrics. Specifically, on the YouTube-VOS dataset, our method improves upon ProPainter by an absolute gain of +0.24 dB in PSNR (from 34.43 dB to 34.67 dB) and +0.012 in SSIM (from 0.974 to 0.986), while also achieving a lower LPIPS score. Similarly, on the DAVIS dataset, our method achieves a PSNR of 34.51 and an SSIM of 0.977, demonstrating consistent advantages, particularly in handling videos with complex dynamic backgrounds. Our approach significantly reduces artifacts and unnatural boundary transitions during inpainting.

These quantitative results validate the effectiveness of our proposed framework CLIP-RL in video inpainting tasks, particularly in terms of image quality and temporal consistency. Unlike traditional fixed-strategy inpainting methods, our approach exhibits adaptability, dynamically adjusting its inpainting strategy based on complex scene characteristics, thereby notably enhancing inpainting performance.

### 4.3. Qualitative Comparisons

**Figure 5:**
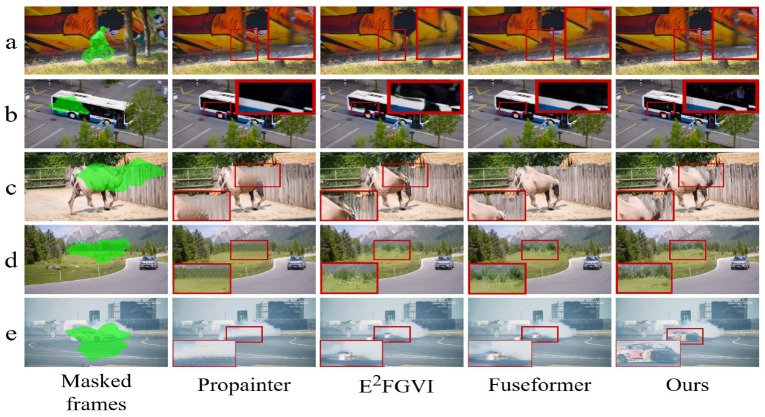
Qualitative comparison of video inpainting across five scenarios: (**a**) object removal, (**b**) simple static background, (**c**) large-area damage, (**d**) complex textures, and (**e**) dynamic backgrounds. The red boxes highlight enlarged areas for detailed comparison. All methods perform well in simple scenarios like (**b**).

In qualitative analysis, we compare our RL-based video inpainting method with existing mainstream approaches, including dual-domain propagation-based ProPainter [11], feature propagation-based E^2^FGVI [5], and Transformer-based FuseFormer [10]. As shown in Figure 5, our method outperforms these baselines across various challenging scenarios.

Notably, in scenes with dynamic backgrounds and complex occlusions—such as those depicted in Figure 5d,e—our method significantly reduces artifacts and ensures smoother boundary transitions. For instance, in the case of large-scale damage shown in Figure 5c, our method reconstructs missing regions with superior fidelity, avoiding the blurring and structural inconsistencies common in baseline methods (e.g., ProPainter exhibits noticeable content inpainting deficiencies and edge artifacts). This superiority is attributed to the dynamic optimization of our RL framework, which adjusts its inpainting strategy based on feedback from the inpainting detection module. Furthermore, as corroborated by the quantitative results in Table 1, our method achieves higher PSNR and SSIM scores in complex scenarios. For example, in dynamic background scenes, our method improves SSIM by 1.2% over the closest competitor, underscoring its effectiveness in maintaining spatial and temporal consistency, as further evidenced by the smooth inter-frame transitions in Figure 5e. However, in simpler inpainting tasks, such as scenarios with minimal damage under static backgrounds (e.g., Figure 5b), performance differences between methods are marginal, as all approaches handle such cases effectively. This highlights our method’s particular strength in complex scenarios, though it may not offer significant advantages in less challenging settings.

Overall, the integration of reinforcement learning and the inpainting detection module enables our framework to deliver high-precision and natural inpainting results in complex application scenarios where existing methods struggle. These qualitative results, supported by quantitative metrics, demonstrate the effectiveness and robustness of our proposed approach.

### 4.4. Ablation Evaluations

#### 4.4.1. Effectiveness of Inpainting Detection Module

**Figure 6:**
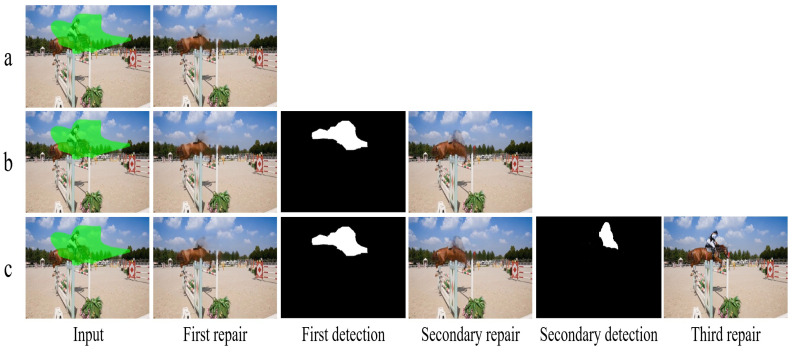
Qualitative results demonstrating the effectiveness of the inpainting detection module in video inpainting. Each row represents: (**a**) no detection module (single-pass inpainting), (**b**) inpainting results after one iteration of detection feedback, and (**c**) inpainting results after two iterations of detection feedback.

To evaluate the contribution of the inpainting detection module to video inpainting performance, we designed an ablation study comparing the performance of the inpainting model with and without the detection module. In these experiments, all configurations excluded the reinforcement learning module. The experimental setup is as follows: in the configuration without the detection module, the inpainting model performs a single round of inpainting; in contrast, with the detection module, the inpainting model refines the results based on one or multiple rounds of feedback from the detection module. The primary function of the detection module is to identify regions with suboptimal inpainting quality and generate predicted masks to guide the inpainting model in refining these regions, thereby reducing artifacts and enhancing visual consistency. As shown in Figure 6, the first row presents the inpainting results without the detection module, where the model fails to fully restore defective regions. The second row displays the results after one round of detection feedback, demonstrating significant improvements in inpainting quality. The third row shows further enhancements after two rounds of detection feedback.

As reported in Table 2, the experimental results indicate that, without the detection module, the inpainting model achieves a PSNR of 32.30 dB and an SSIM of 0.955. With the detection module and one round of feedback, the PSNR improves to 34.16 dB and the SSIM to 0.971. After two rounds of feedback, the PSNR reaches 34.57 dB and the SSIM 0.978. These results validate the effectiveness of incorporating a detection mechanism for feedback in video inpainting tasks, particularly in complex scenes, where it significantly enhances the visual consistency and structural integrity of the inpainting results. Moreover, the progressive improvement observed across multiple feedback iterations suggests that our detection-guided refinement strategy can effectively mitigate accumulated detection errors, as inaccurate or uncertain regions are re-evaluated and corrected in subsequent rounds. This iterative process enhances the robustness and stability of the overall framework, ensuring consistent performance even under imperfect detection conditions.

#### 4.4.2. Effectiveness of the Reinforcement Learning Module

To evaluate the role of the reinforcement learning module in the proposed CLIP-RL framework, this experiment compared the performance of the model without the reinforcement learning module (0 iterations) against different iteration counts (1 to 5), recording computational cost (GFLOPs) and inpainting quality (PSNR, SSIM). Table 3 shows that without the reinforcement learning module, the PSNR was 34.43 dB and SSIM was 0.974. After incorporating the reinforcement learning module, both PSNR and SSIM improved with increasing iterations, reaching 34.67 dB (an improvement of 0.24 dB) and 0.986, respectively, at 3 iterations.

**Figure 7:**
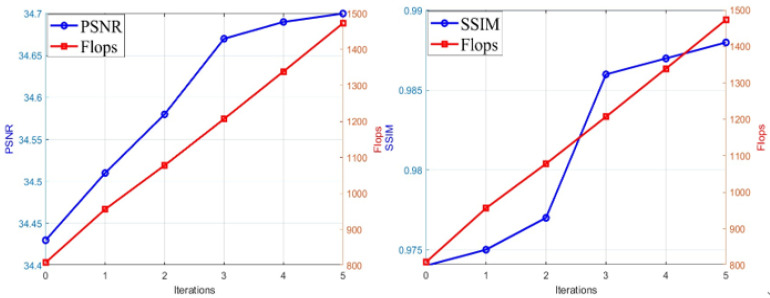
Impact of Reinforcement Learning Iteration Count on PSNR, SSIM, and GFLOPs.

Figure 7 further illustrates the relationship between iteration count and PSNR, SSIM, and Flops. The curves show that PSNR, SSIM, and Flops exhibit a positive correlation with the number of iterations. While continuing to increase the number of iterations beyond three results in marginal improvements, the associated computational cost also increases, and the gains in inpainting quality begin to diminish. This suggests that although additional iterations improve inpainting quality, the computational overhead leads to diminishing returns. Thus, an iteration count of three achieves the best cost-performance balance.

Although the reinforcement learning module inevitably increases computational cost (from 808 to 1207 GFLOPs at three iterations), it substantially enhances the model’s robustness and ensures stable, coherent inpainting results across diverse and dynamic scenes. By explicitly modeling temporal dependencies and motion variations, the module improves visual consistency under challenging conditions. This moderate increase in complexity represents a reasonable trade-off between performance and efficiency, while the subsequent dynamically adjustable attention-head mechanism further compensates for the computational overhead introduced by reinforcement learning.

### 4.5. Analysis of Reinforcement Learning Actions and Loss Optimization

#### 4.5.1. Analysis of Dynamic Attention Head Optimization

To verify the adaptive capability of the dynamic adjustment mechanism for the number of attention heads ($h_{\mathrm{head}}$) in the CLIP-RL framework, we designed ablation experiments on the DAVIS validation set, comprising two task categories: easy inpainting tasks (static backgrounds) and hard inpainting tasks (dynamic backgrounds), each with 50 video samples. The experiments compared fixed head configurations ($h_{\mathrm{head}}=4$ and $h_{\mathrm{head}}=8$) with the dynamic adjustment mechanism (up to 12 heads), evaluating inpainting quality using PSNR and SSIM, and efficiency using per-frame inference time.

Table 4 shows that, for easy tasks, increasing the fixed head count from 4 to 8 improves PSNR from 34.46 dB to 34.51 dB (a gain of 0.05 dB) and SSIM from 0.968 to 0.974 (a gain of 0.006), while inference time rises from 0.091 s/frame to 0.094 s/frame (a 3.3% increase). This indicates that 4 heads are sufficient for static backgrounds, with limited benefits from additional heads. The dynamic adjustment mechanism, activating an average of 5.88 heads, achieves a PSNR of 34.49 dB (0.03 dB higher than 4 heads) and an SSIM of 0.972, with an inference time equivalent to 4 heads (0.091 s/frame), balancing efficiency and performance. For hard tasks, the fixed 8-head configuration improves PSNR by 2.04 dB (from 32.03 dB to 34.07 dB) and SSIM by 0.019 (from 0.947 to 0.966) compared to 4 heads, with an inference time of 0.092 s/frame. The dynamic adjustment mechanism, activating an average of 9.72 heads, achieves a PSNR of 34.33 dB and an SSIM of 0.971, surpassing the 8-head configuration by 0.26 dB and 0.005.

In summary, the dynamic attention-head selection mechanism adaptively adjusts the number of heads according to task complexity—reducing computation for simple scenes while activating more capacity for challenging ones. This adaptive strategy effectively balances inpainting quality and efficiency, maintaining high reconstruction fidelity with minimal additional cost and mitigating the computational burden introduced by reinforcement learning iterations.

#### 4.5.2. Analysis of Dynamic Fusion Strategy Optimization

To validate the contribution of the dynamic fusion strategy, which adaptively adjusts the fusion strength $w_{\mathrm{fuse}}\in \left[0.5,0.8\right]$ via reinforcement learning (RL) in terms of boundary smoothness and artifact reduction, particularly in scenarios involving fast motion and large-area occlusions, we designed ablation experiments on the DAVIS validation set. These experiments encompassed two types of scenarios: rapidly changing backgrounds and large-area missing regions, each comprising 50 video samples. We compared three configurations: (1) Dynamic Fusion (Ours): The RL agent adjusts $w_{\mathrm{fuse}}$ based on the state (inpainted frame features, FOCAL mask), with other actions (e.g., number of attention heads, reward weights α, β, γ) fixed; (2) Fixed Fusion: A constant $w_{\mathrm{fuse}}=0.65$; (3) No Fusion: Direct use of the inpainted frame ($w_{\mathrm{fuse}}=1$). Performance was evaluated using Gradient Matching Score (GMS), Edge Similarity (ES), Peak Signal-to-Noise Ratio (PSNR), and Structural Similarity Index (SSIM).

Table 5 presents the experimental results for the fast-motion and large-area occlusion scenarios. The dynamic fusion strategy achieved the best overall performance, with PSNR values of 34.50 and 34.51 and SSIM values of 0.978 in both scenarios. In terms of boundary consistency, the dynamic fusion approach yielded GMS scores of 0.94 and 0.93 and ES scores of 0.89 and 0.88, significantly outperforming the fixed fusion and no fusion configurations. These results demonstrate the marked advantage of dynamic fusion in fast-motion and large-area occlusion scenarios, particularly in enhancing boundary consistency. Although the dynamic fusion strategy increased inference time and computational cost (0.101 seconds per frame), its substantial improvements in inpainting quality, boundary consistency, and perceptual quality make it particularly suitable for high-quality video inpainting tasks.

#### 4.5.3. Analysis of Reward Weight Evolution and Mechanism Effectiveness

To evaluate the necessity of the proposed dynamic reward weight adjustment mechanism and understand its optimization dynamics under multi-objective constraints, we conducted a comparative analysis against fixed strategies and visualized the weight evolution across iterative refinement stages. We established two baselines on the DAVIS validation set: (1) Fixed-Bias (α=1.0, β=0.3, γ=0.1), which theoretically maximizes immediate numeric rewards but minimizes constraint penalties, serving as a proxy to test susceptibility to trivial solutions (i.e., “reward hacking”); and (2) Fixed-Average (α=0.9, β=0.45, γ=0.2), representing a static compromise between conflicting objectives.

Table 6 presents the quantitative performance of these strategies. The Fixed-Bias configuration yields the poorest performance (33.92 dB PSNR), indicating that naively prioritizing easier metrics fails to drive effective spatiotemporal recovery. While the Fixed-Average setting improves performance to 34.45 dB, it remains suboptimal. In contrast, our Dynamic strategy achieves the highest PSNR (34.51 dB) and SSIM (0.977), along with the lowest warping error ($E_{warp}=1.002$). These results demonstrate that the dynamic mechanism effectively navigates the complex optimization landscape, outperforming static strategies by adaptively rebalancing objectives during inference.

**Figure 8:**
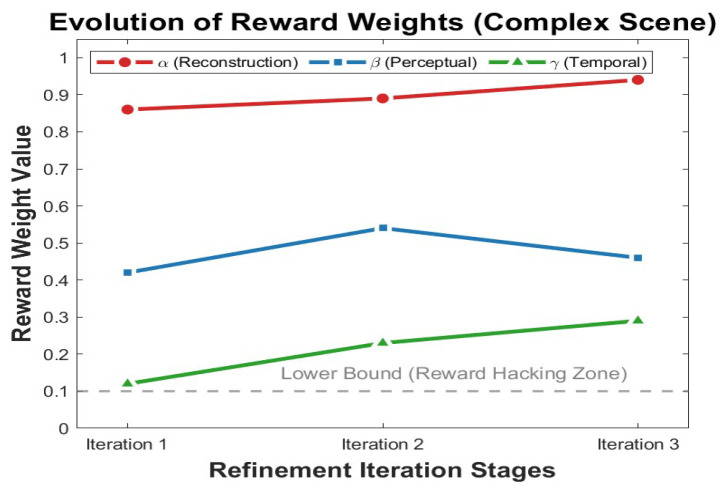
Evolutionary trajectory of reward weights and analysis of the agent’s learned optimization strategy across three refinement iterations. The data demonstrates the dynamic balancing of reconstruction (α), perceptual quality (β), and temporal consistency (γ) to achieve optimal spatiotemporal coherence.

Figure 8 visualizes the trajectory of reward weights (α,β,γ) across three refinement iterations, revealing an intrinsic “coarse-to-fine” optimization curriculum learned by the agent. Specifically, in the first iteration, the temporal weight γ (green line) starts low (0.12), a conservative initialization that mitigates the risk of error propagation caused by unreliable optical flow estimation in the early stages. Moving to the second iteration, a distinct peak in the perceptual weight β (0.54) is observed, indicating a strategic shift towards texture hallucination and high-frequency detail recovery. Finally, in the third iteration, the agent voluntarily escalates γ to its maximum (0.29), enforcing strict temporal alignment to rectify inter-frame jitter and ensuring global coherence.

This evolutionary pattern confirms that the proposed mechanism effectively prevents the model from converging to local optima or exploiting easier reward terms at the expense of critical constraints. By autonomously increasing the weight of the challenging temporal alignment term (γ) in later stages, the agent demonstrates a robust preference for long-term video quality over short-term numeric gains. While this dynamic process introduces computational complexity, it significantly bolsters training stability and ensures superior convergence in complex scenarios.

## 5. Conclusions

This study presents CLIP-RL, a closed-loop video inpainting framework that employs detection-guided reinforcement learning for adaptive strategy optimization. Extensive experiments demonstrate that CLIP-RL achieves superior visual quality and temporal consistency compared with existing methods. The proposed architecture-agnostic paradigm can be readily adapted to other backbones by redefining relevant hyperparameters—such as receptive fields or propagation iterations—as agent actions. Moreover, the detection-guided adaptive optimization concept shows strong potential for broader vision tasks, particularly in enhancing robustness for visual tracking by addressing challenges like occlusion and domain shifts [32,33,34].

Despite its effectiveness, certain limitations remain. CLIP-RL’s performance declines under extreme long-term occlusions or abrupt scene transitions, where temporal cues are severely disrupted, revealing that its temporal modeling of long-range dependencies remains limited. In addition, the iterative RL process introduces a moderate computational overhead while ensuring stable performance. Future work will focus on investigating stronger temporal constraints or loss formulations to further enhance temporal consistency, while also exploring more efficient optimization strategies to reduce computational complexity.

## Figures and Tables

**Table 1:** Quantitative comparison on YouTube-VOS and DAVIS datasets. ↑ (↓) indicates that higher (lower) is better. The best results are highlighted in bold. Publication year is indicated for each compared method to highlight temporal coverage.

Method	Year	YouTube-VOS			DAVIS Square			DAVIS Object		
		PSNR ↑	SSIM ↑	LPIPS ↓	PSNR ↑	SSIM ↑	LPIPS ↓	PSNR ↑	SSIM ↑	LPIPS ↓
VINet	2019	29.83	0.955	0.047	28.32	0.943	0.049	28.47	0.922	0.083
DFGVI	2019	32.05	0.965	0.038	29.75	0.959	0.037	30.28	0.925	0.052
CPN	2019	32.17	0.963	0.040	30.20	0.953	0.049	31.59	0.933	0.058
OPN	2019	32.66	0.965	0.039	31.15	0.958	0.044	32.40	0.944	0.041
3DGC	2019	30.22	0.961	0.041	28.19	0.944	0.049	31.69	0.940	0.054
STTN	2020	32.49	0.964	0.040	30.54	0.954	0.047	32.83	0.943	0.052
FGVC	2020	33.94	0.972	0.026	32.14	0.967	0.030	33.91	0.955	0.036
TSAM	2021	31.62	0.962	0.031	29.73	0.951	0.036	31.50	0.934	0.048
FFM	2021	33.73	0.970	0.030	31.87	0.965	0.034	34.19	0.951	0.045
FGT	2022	32.17	0.960	0.028	32.60	0.965	0.032	34.30	0.953	0.040
LNFVI	2024	30.80	0.970	**0.025**	31.35	0.957	0.027	–	–	–
ProPainter	2023	34.43	0.974	0.033	34.47	**0.978**	0.035	34.45	0.975	0.036
**Ours**	**2025**	**34.67**	**0.986**	0.031	**34.51**	0.977	**0.030**	**34.50**	**0.979**	**0.033**

**Table 2:** Ablation study results of the detection module.

Configuration	PSNR	SSIM	Inference Time (s/Frame)
Without detection module	32.30	0.955	0.083
With detection module (1 feedback)	34.16	0.971	0.088
With detection module (2 feedbacks)	34.57	0.978	0.091

**Table 3:** Impact of RL module iteration count on computational cost and inpainting quality. The checkmark (✓) indicates that the RL module is enabled, and the upward arrow (↑) denotes that higher values represent better performance.

RL Module	—	✓	✓	✓	✓	✓
Iterations	0	1	2	3	4	5
GFLOPs	808	956	1078	1207	1339	1473
PSNR ↑	34.43	34.51	34.58	34.67	34.69	34.70
SSIM ↑	0.974	0.975	0.977	0.986	0.987	0.988

**Table 4:** Ablation results of dynamic $h_{\mathrm{head}}$ under two scenarios.

Scenario	Method	Max $h_{\mathrm{head}}$	Avg. Active Heads	PSNR	SSIM	Inference Time (s/Frame)
Easy inpainting	Fixed	4	4	34.46	0.968	0.091
	Fixed	8	8	34.51	0.974	0.094
	Ours	12	5.88	34.49	0.972	0.091
Hard inpainting	Fixed	4	4	32.03	0.947	0.091
	Fixed	8	8	34.07	0.966	0.092
	Ours	12	9.72	34.33	0.971	0.094

**Table 5:** Ablation results of dynamic fusion under two scenarios.

Scenario	Method	PSNR	SSIM	GMS	ES	Inference Time (s/Frame)
Fast Background Change	No Fusion	34.10	0.968	0.84	0.79	0.091
	Fixed Fusion	34.35	0.973	0.89	0.84	0.091
	Dynamic Fusion (Ours)	34.50	0.978	0.94	0.89	0.101
Large-Area Missing	No Fusion	34.05	0.965	0.83	0.78	0.091
	Fixed Fusion	34.39	0.970	0.88	0.83	0.091
	Dynamic Fusion (Ours)	34.51	0.978	0.93	0.88	0.101

**Table 6:** Quantitative comparison of reward weight strategies on the DAVIS validation set. The proposed Dynamic strategy is evaluated against fixed baselines to verify mechanism effectiveness. Best results are in bold. ↑ indicates that higher values are better; ↓ indicates that lower values are better.

Strategy	Configuration (α,β,γ)	PSNR ↑	SSIM ↑	$E_{\mathrm{warp}}$↓
Fixed-Bias	Fixed at bounds (1.0,0.3,0.1)	33.92	0.965	1.048
Fixed-Average	Fixed at mean (0.9,0.45,0.2)	34.45	0.975	1.012
Dynamic (Ours)	Adaptive adjustment	**34.51**	**0.977**	**1.002**

## Data Availability

Publicly available datasets were analyzed in this study. These data can be found here: YouTube-VOS: https://youtube-vos.org/dataset/ (accessed on 31 October 2025); DAVIS: https://davischallenge.org/davis2017/code.html (accessed on 31 October 2025).
